# Characterisation of an Anti-Vaccinia Virus F13 Single Chain Fragment Variable from a Human Anti-Vaccinia Virus-Specific Recombinant Immunoglobulin Library

**DOI:** 10.3390/v14020197

**Published:** 2022-01-20

**Authors:** Henrike P. Ahsendorf, Ulrike S. Diesterbeck, Sven-Kevin Hotop, Michael Winkler, Mark Brönstrup, Claus-Peter Czerny

**Affiliations:** 1 Division of Microbiology and Animal Hygiene, Department of Animal Sciences, University of Göttingen, Burckhardtweg 2, 37077 Göttingen, Germany; henrike.ahsendorf@arcor.de (H.P.A.); cczerny@gwdg.de (C.-P.C.); 2 Helmholtz Centre for Infection Research, Inhoffenstraβe 7, 38124 Braunschweig, Germany; Sven-Kevin.Hotop@helmholtz-hzi.de (S.-K.H.); Mark.Broenstrup@helmholtz-hzi.de (M.B.); 3 Infection Biology Unit, German Primate Center, Leibniz Institute for Primate Research, Kellnerweg 4, 37077 Göttingen, Germany; MWinkler@dpz.eu

**Keywords:** scFv, vaccinia virus, recombinant antibody, F13, epitope mapping

## Abstract

Vaccinia virus (VACV) belongs to the genus Orthopoxvirus of the family Poxviridae. There are four different forms of infectious virus particles: intracellular mature virus (IMV), intracellular en-veloped virus (IEV), cell-associated enveloped virus (CEV) and extracellular enveloped virus (EEV). The F13 protein occupies the inner side of the CEV- and EEV-membranes and the outer side of the IEV-membranes. It plays an important role in wrapping progress and EEV production. We constructed a human single-chain fragment variable (scFv) library with a diversity of ≥4 × 108 independent colonies using peripheral blood from four vaccinated donors. One anti-F13 scFv was isolated and characterised after three rounds of panning. In Western blotting assays, the scFv 3E2 reacted with the recombinant F13VACV protein with a reduction of binding under denatured and reduced conditions. Two antigenic binding sites (139-GSIHTIKTLGVYSDY-153 and 169-AFNSAKNSWLNL-188) of scFv 3E2 were mapped using a cellulose membrane encompassing 372 15-mere peptides with 12 overlaps covering the whole F13 protein. No neutralisation capa-bilities were observed either in the presence or absence of complement. In conclusion, the con-struction of recombinant immunoglobulin libraries is a promising strategy to isolate specific scFvs to enable the study of the host-pathogen interaction.

## 1. Introduction

Vaccinia virus (VACV), a member of the genus *Orthopoxvirus* (OPXV) of the family *Poxviridae* [1], was used as the vaccine, which led to the eradication of smallpox in 1979 [2]. While variola virus (VARV) solely infects humans, OPXV with zoonotic potential, like cowpox virus (CPXV) and monkeypox virus (MPXV), can also cause severe and sometimes fatal infections [3,4,5,6,7,8,9]. There are public concerns about bioterrorism using poxviruses as biological weapons [10]. Therefore, the investigation of poxvirus replication and infectivity is still necessary.

There are four different types of infectious virus particles: intracellular mature virus (IMV), intracellular enveloped virus (IEV), cell-associated enveloped virus (CEV) and extracellular enveloped virus (EEV) [11,12]. The majority of the particles are IMV virions (>90%), which are responsible for the transmission of the virus between hosts and are generated within cytoplasmic factories from crescents precursor cells [13,14]. Some IEV particles get out of the factories and receive a double layer of the intracellular membrane by the trans-Golgi network (TGN) or the early endosomes [15]. The outer membrane of IEV fuses with the cell’s plasma membrane [16]. The particles stay connected to the cell surface named CEV, while the detached ones are termed EEV [12,17]. The CEV, as well as the EEV, are responsible for a rapid virus spread within the host [13]. Each form has a unique antigen occupancy and distribution on its surface [1,12,18,19,20,21,22,23,24,25,26,27]. For instance, the major envelope protein of EEV particles in the 37 kDa F13 non-glycosylated membrane protein [28,29], encoded by the ORF F13L gene and consists of 372 aa [29,30]. The F13 protein has no transmembrane domain, but it is palmitoylated at cysteine residues 185 and 186 [31], which are located within the TGN membrane [32]. The F13 plays an important role in the membrane association, the virion wrapping progress and the EEV production [33,34].

Although smallpox has been eradicated, there is a rising interest in neutralising antibodies as well as antiviral drugs because of the fear of bioterrorism [35,36]. The generation of highly diverse species-specific human antibody libraries by using the phage display technique [37] is a powerful technology. Target-specific human single-chain variable antibody fragments (scFvs) can be even used as a treatment because they can penetrate the cell due to their low molecular weight [38].

In this study, we constructed an anti-F13_VACV_ scFv antibody retrieved from a human immunoglobulin library isolated from an OPXV vaccine. The specificity, binding affinity and virus neutralisation capacities of the F13 scFv were compared to that of a monoclonal antibody.

## 2. Material and Methods

### 2.1. Cells and Viruses

The permanent monkey kidney cell line MA-104 cultured in minimum essential medium (MEM) and supplemented with 7% foetal calf serum was used to propagate the VACV strains Elstree and Munich 1 [39]. Infectivity titres were determined and calculated as plaque-forming units (pfu/mL). Vero cells cultured in MEM and supplemented with 5% foetal calf serum were used for plaque reduction tests.

Virus preparations were purified and concentrated by sucrose gradient centrifugation as described previously [40,41]. The protein contents of the samples were determined by the method of Lowry et al. [42].

### 2.2. Monoclonal and Polyclonal Antibodies

For this study, the A27-specific murine mAb anti-VACV 5B4/2F2 (epitope #1A) [39,41] and the rat mAb 15B6 directed against the VACV envelope protein F13 [43,44] were used. The mAb 15B6, used as F13 positive control, was kindly made available by Jacomina Krijnse Locker. An anti-his-tag antibody (Qiagen, Hilden, Germany) was used to evaluate the preparation of the protein purifications. Moreover, polyclonal rabbit hyper-immune serum against purified A27_VACV_ [39,41], used in the confocal experiment, was purified on Protein G Sepharose columns (HiTrap ™ 5 mL Protein G HP, Sigma Aldrich, USA), dialyzed against PBS and sterilised by centrifugation at 20,238× *g*. Protein contents of all antibodies preparations were determined according to the method of Lowry et al. [42].

### 2.3. Immunisation, Lymphocyte Preparation and Library Construction

Four human volunteers were immunised via scarification with Dryvax^®^ (Wyeth Laboratories, Marietta, GA, USA) according to the manufacturer’s instructions as described before [45]. 20–28 days post-vaccination, about 500 mL blood was collected, the peripheral blood mononuclear cells were isolated using the Ficoll-Paque PLUS density gradient (GE Bioscience, Freiburg, Germany), followed by RNA extraction (RNeasy MiniKit (Qiagen, Hilden, Germany)) and by cDNA synthesis using oligohexamers (pdN_6_) (Invitrogen, Karlsruhe, Germany) as directed by the manufacturers. To construct the scFv library, RT-PCR was performed using total RNA of at least 10^7^ cells per volunteer as described before [45].

### 2.4. Construction and Purification of F13

For the amplification of the F13L gene and its truncated sub-fragment, primers were selected by using a published sequence (GenBank accession number: NC_006998.1). The restriction enzymes *Bam*HI and *Hind*III were introduced to both ends of the primers. The reserve primer “F13 (C-terminal)” 5′-GAT CAA GCT TTT AAA TTT TTA ACG ATT TAC-3′, and the following forward primers were used to amplify the full-size F13 protein and the truncated fragment #1: “F13 forward (N-terminal)”: 5′-GAT CGG ATC CAT GTG GCC ATT TGC ATC GG-3′, “fragment #1 (N-terminal)”: 5′-GAT CGG ATC CAG AAT CCT ATA GGT GGA GTG-3′. The PCR reactions were set as follows: initial denaturation at 95 °C/10 min, 35 cycles of 94 °C/1 min, primer annealing at 50.6 °C/1 min and 72 °C/2 min, and a final extension step of 72 °C/10 min.

The PCR products were ligated into the pSC-A-amp/kan PCR cloning vector (StrataClone, Agilent Technologies, Boeblingen, Germany) and transformed into chemically competent *E. coli*. Ligation and transformation were performed according to the manufacturer. Plasmids of positive colonies were isolated from 5 mL LB media using the MiniPrep Kit (Qiagen, Hilden, Germany). The genes were sequenced with an ABI Prism 3100 Analyzer (Applied Biosystems Deutschland GmbH, Darmstadt, Germany). Sequences were analysed using the DNAStar program (SeqMan Pro and MegAlign. Version 12.0. DNASTAR. Madison, WI, USA) and BLAST [46].

Plasmids were ligated into the expression vectors pQE80L and pQE81L (Qiagen, Hilden, Germany), electroporated into One Shot ^®^ TOP10 Electrocomp™ *E. coli*. (Invitrogen ^TM^, Karlsruhe, Germany) and were grown in LB media with 1 mM Ampicillin at 37 °C until OD_600 nm_ = 0.6. The expression was induced with 1 mM IPTG at 37 °C for 5 h while shaking at 200 rpm (Sartorius Certomat ^®^ BS-1, Göttingen, Germany). Cells were pelleted (4500 rpm/20 min) and resuspended in TBS buffer containing 7.7 mM Tris pH 7.5/150 mM NaCl before sonicated (100% on ice/ 20 min). Cell debris was removed by centrifugation (2100 rpm/15 min). The lysate was pelleted at 7000 rpm for 1 h and resuspended in suspension buffer containing 12.5% phosphate buffer/1% 2 M imidazole stock/23% of 43.5% glycerol stock at 4 °C overnight. Afterwards, the lysates were mixed with an equal volume of 16 M urea stock and incubated by gently shaking at 4 °C for 1 h. Lysates were clarified at 17,000× *g* at 4 °C for 45 min. Supernatants were compounded with Ni-NTA agarose (Qiagen, Hilden, Germany) and incubated under gently shaking at 4 °C overnight. The mixtures were loaded onto 5 mL Ni-NTA columns (Qiagen, Hilden, Germany). The purifications were done according to the manufacturer instructions by using a binding buffer pH 8.0 containing 12.5% phosphate buffer/1% 2 M imidazole stock/23% of 43.5% glycerol stock/50% 16 M urea stock and an elution buffer containing 12.5% phosphate buffer/5% 2 M imidazole stock/23% of 43.5% glycerol stock/50% 16M urea stock. After overnight dialysis against PBS, the protein concentrations were determined by Lowry protein assay as described previously [42].

### 2.5. Selection of Purified Recombinant F13 Protein

To get specific anti-F13 scFv antibodies, the constructed human anti-OPXV-scFv phage library was panned three times, applying purified recombinant F13 protein of VACV Munich 1. The exact screening procedure was performed as previously described [45].

### 2.6. Plasmid Isolation and Sequencing of Positive Colonies

To produce antibody fragments without pIII fusion, 0.5 µL of the *E. coli* HB2151 pre-cultures were transferred into 100 µL 2 × TYG (0.1%)-A and incubated at 30 °C for 4 h. The expression was induced by the addition of IPTG to a final concentration of 2 mM dissolved in 50 µL 2 × TY-A by gentle shaking at 30 °C overnight (Sartorius Certomat ^®^ BS-1, Göttingen, Germany). The cells were centrifuged (137,000× *g*/4 °C/20 min) and the supernatants were applied in an ELISA for pre-screening as described before [45]. The positive plasmid was isolated from 5 mL media using the MiniPrep Kit (Qiagen, Hilden, Germany). With an ABI Prism 3100 Analyzer (Applied Biosystems Deutschland GmbH, Darmstadt, Germany), the genes encoding the variable regions of the heavy (VH) and light (VL) chains were sequenced. Therefore, the vector-specific forward primer R1 (5′-CCA TGA TTA CGC CAA GCT TTG GAG CC-3′) and the reverse primer R2 (5′-CGA TCT AAA GTT TTG TCG TCT TTC C-3′) were applied to the sequence reaction. The sequence was analysed with the DNAStar program (SeqMan Pro and MegAlign. Version 12.0. DNASTAR. Madison, WI). Moreover, the amino acid sequence was used to classify the presumed family and germline origin by searching IMGT/V-QUEST [47,48] and IMGT/DomainGapAlign. Furthermore, the structure of the scFv was analysed and a 3D model was calculated using the Phyre^2^ server [49] and VMD 1.9.1 software [50].

### 2.7. Upscale Production and Purification of Selected scFv

Production of the scFv yielding the highest ELISA value was scaled up to one litre and the antibody was purified as described before [45]. Finally, the Lowry protein assay determined the protein concentration as described previously [42].

### 2.8. SDS-PAGE and Western Blotting

For Western blotting analyses, 5 µg/slot of gradient purified VACV Elstree, gradient purified VACV Munich1, purified recombinant A27 protein and of the purified recombinant F13 protein were fractionated by vertical 12% sodium dodecyl sulphate (SDS)-polyacrylamide gel electrophoresis [51] and subsequently transferred to nitrocellulose membranes [5]. The blocking step was performed by a mixture of 3% BSA in TBS at room temperature for 2 h. Purified Abs (50 µg/mL) were added to the membranes and incubated at room temperature for 2 h. Immunodetection followed with horseradish peroxidase-conjugated anti-IgG antisera (1:250) for 2.5 h and horseradish peroxidase colour-developing reagent (Bio-Rad, Heidelberg, Germany; 375 µg/mL). The membranes were washed with TBS three times for 10 min between each step. The Mr of stained viral proteins was estimated with a concurrent protein standard (Bio-Rad, Munich, Germany).

### 2.9. Enzyme-Linked Immunosorbent Assay (ELISA)

Microtiter plates were coated with either of 2 µg/mL VACV Elstree, 2 µg/mL VACV Munich1, 15 µg/mL F13_VACV_, 5 µg/mL A27_VACV_, and with 2 µg/mL BSA. After blocking, antibodies were added in two-fold serial dilutions starting with a concentration of 200 µg/mL. Incubation was performed at 37 °C for 3 h. After washing with PBS five times, the detection of the second antibody occurred either with goat pAb to E tag (HRP) (1:2000), anti-mouse IgG (whole molecule) or anti-rat IgG (whole molecule) peroxidase conjugate developed in goat (1:2000) at 37 °C for 1 h. After washing with PBS ten times, the developing solution composed of tetramethylbenzidine (TMB) was added. The reaction development was stopped using hydrochloric acid. The photometric reading (Spectra II, SLT Labinstruments GmbH, Germany) was performed at 450 nm. The binding affinity was calculated from the average adsorption of three independent assays using the Michaelis–Menten behaviour [52] using GraphPad Prism version 6.00 for Mac (La Jolla, CA, USA).

### 2.10. Epitope Mapping by SPOT Synthesis on Nitrocellulose Membranes

A total of 372 amino acids representing whole F13 protein were synthesised directly on derivatised cellulose membranes in the form of 15-mere peptides with 12 aa overlaps. The synthesis of derivatised cellulose membranes was performed as described before [53]. The protein binding assay, as well as the subsequent membrane regeneration, were executed as previously described [54].

### 2.11. Cell Infection and Confocal Microscopy

A confluent monolayer of vero cells was cultivated on glass coverslips placed in 24-well tissue culture plates. Then, 100 pfu (100 µL/well) of VACV Munich1 was added to each well. After incubation at 37 °C for 3 h, the virus mixture was replaced with a fresh medium containing 2.5% FCS. Cells were incubated at 37 °C for about 18 h, followed by removing the medium. Cells were fixed on glass coverslips with 4% paraformaldehyde in PBS at room temperature for 20 min. After that, 0.2% Triton X-100 in PBS was added and incubated at room temperature for 5 min to permeabilise the cells. After three washing-steps with PBS, cells were blocked with PBS supplemented with 10% FCS (200 µL/well) at room temperature for 30 min. Subsequently, the blocking solution was removed and 100 µg/mL of the primary Abs diluted in PBS supplemented with 10% FCS (250 µL/well) were incubated at 4 °C for 2 h, followed by further 30 min at room temperature. After three washing-steps with PBS, both the Hoechst’ reagent (1:5000; 100 µL/well) to stain the cell nuclei and the secondary Abs (1:1000 goat anti-mouse Alexa Fluor 488 or goat anti-rabbit Alexa Fluor 568 (Abcam, Cambridge, UK) diluted in PBS containing 10% FCS (250 µL/well)) were added and incubated at room temperature for 1 h. After three washing steps with PBS, the glass coverslips were mounted in Mowiol/DABCO. Fluorescence was examined on a confocal laser scanning microscope (LSM 800, Zeiss) equipped with a Plan-Apochromat 63×/1.40 Oil DIC objective.

### 2.12. In Vitro Plaque Reduction Neutralisation Test (PRT)

To assess the neutralisation abilities of selected antibodies, a confluent monolayer of vero cells was grown in 24-well tissue culture plates. The PRT was performed as described before [55]. Plaques were counted by visual inspection. Neutralisation was determined as ≥50% plaque reduction compared to the virus control.

## 3. Results

### 3.1. Selection of an Anti-F13 Specific scFv Antibody

**Figure 1:**
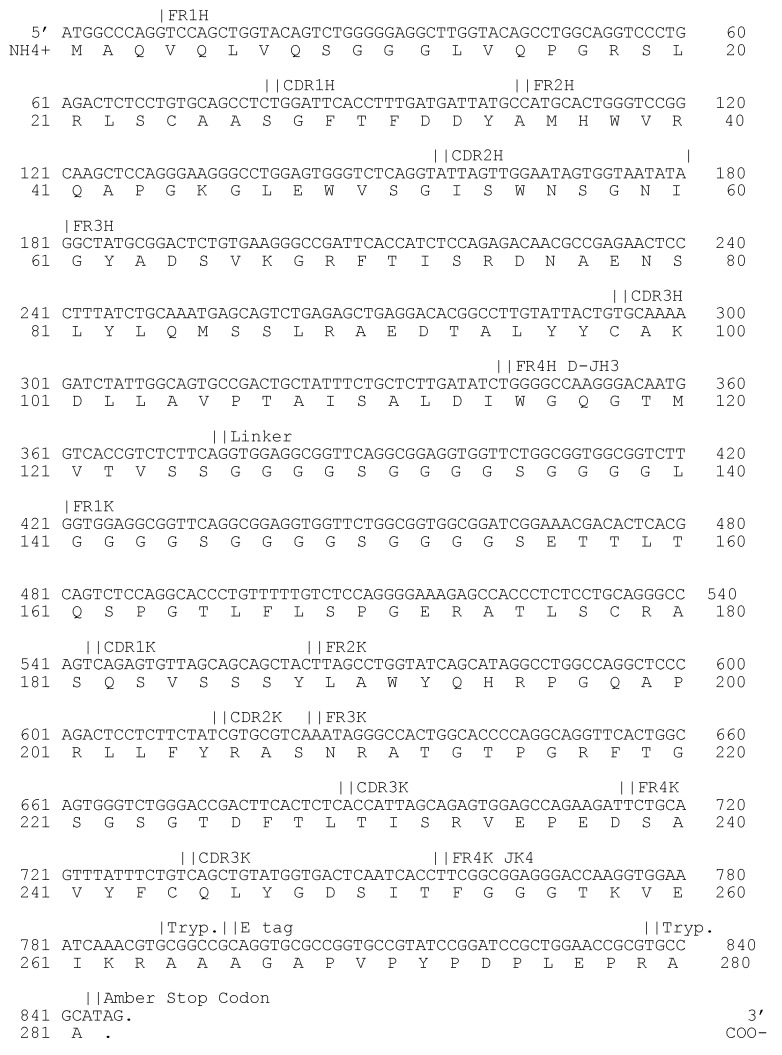
Genome sequence (upper lines) and amino acid sequence (lower lines) of variable domains of heavy (H) and kappa light (κ) chain of the scFv 3E2. The variable region consists of four framework regions (FR1-4) and three hypervariable complementarity-determining regions (CDR1-3). Trypsin-sensitive sites (Tryp.) are needed for the elution of bound phages by enzymatic cleavage with trypsin.

The selection of F13 specific antibodies was conducted over three rounds of panning. After each round, 176 individual *E. coli* HB2151 colonies were isolated to produce soluble antibodies in a microtiter well format. No specific binding of scFv was observed after the first and second selection rounds. The third selection round revealed one clone with an adsorption three times over the background designated as 3E2. The scFv 3E2 (Acc. No.: MW520862) was sequenced and classified to the human VH3/D2/JH3-VK3/JK4 families (Figure 1).

### 3.2. Specificity and Binding Affinity Studies

**Figure 2:**
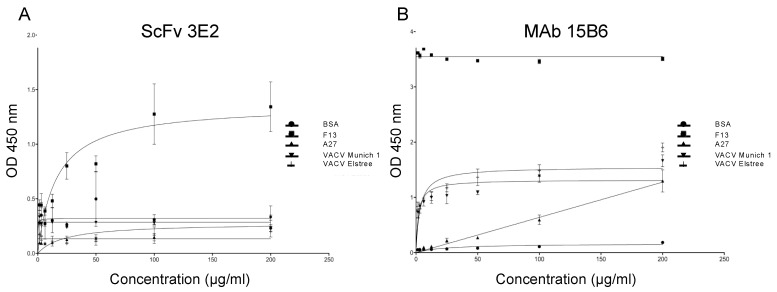
Binding affinities of the scFv 3E2 (**A**) and mAb 15B6 (**B**) as measured in an indirect ELISA using recombinant F13, A27 proteins of VACV, VACV Elstree, Munich1 or BSA. The scFv 3E2 showed a reaction with the recombinant F13 protein. However, the Ab is neither bound to VACV Elstree, VACV Munich1 nor to BSA and A27; the latter were used as negative controls. The mAb 15B6 showed a reaction with the recombinant F13 protein, VACV Elstree and Munich1, while no binding was observed with the recombinant A27 protein and with BSA.

The specificity of the antibodies was measured in an indirect ELISA with two-fold serial dilutions of the scFv starting with 200 µg/mL using various recombinant antigens and viruses (recombinant F13 and A27 proteins of VACV, VACV Elstree and Munich1). The binding affinities were calculated according to Michaelis–Menten [52] (Figure 2).

The scFv 3E2 did not bind to VACV Elstree, VACV Munich1 and the recombinant A27; however, a Michaelis–Menten constant (K_m_) of 10.97 ng/mL and a maximal velocity (v_max_) of 1.25 were recorded against the F13 protein (Figure 2A). For comparison, the mAb 15B6 showed a K_m_ of 0.006724 ng/mL and a v_max_ of 3.54 against the recombinant F13 protein, a K_m_ of 0.7522 ng/mL and a v_max_ of 1.35 towards VACV-Elstree and a K_m_ of 0.5108 ng/mL and a v_max_ of 1.19 towards VACV-M1. The mAb 15B6 did not bind to the recombinant A27 (Figure 2B).

**Figure 3:**
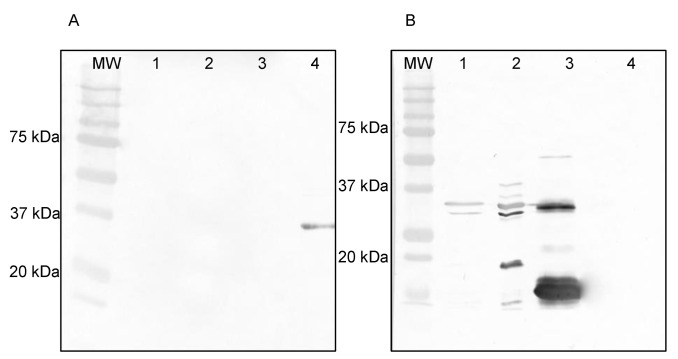
Western blotting analysis of the scFv 3E2 (**A**) and the mAb 5B4/2F2 (**B**) under reducing and denaturing conditions. The scFv 3E2 bound to its epitope on the recombinant F13 protein (Lane 4), however not on VACV Elstree (Lane 1), VACV Munich1 (Lane 2) or on the A27 protein (Lane 3), which was used as a negative control. The mAb 5B4/2F2 showed a reaction on VACV Elstree (Lane 1), VACV Munich1 (Lane 2) and on the recombinant A27 protein of VACV (Lane 3).

Western blotting analysis revealed the binding of the scFv 3E2 on the recombinant F13 protein of VACV under denaturing and reducing conditions (Figure 3A), while no reaction was observed with the VACV Elstree, VACV Munich1 and the A27 protein. As the antibody-positive control, the mAb 5B4/2F2, directed against the A27 protein, was used and showed reactivities with VACV Elstree, VACV Munich1 and the recombinant A27 protein of VACV (Figure 3B).

### 3.3. Epitope Mapping Using a Peptide Membrane and Truncated Recombinant F13 Fragment

**Figure 4:**
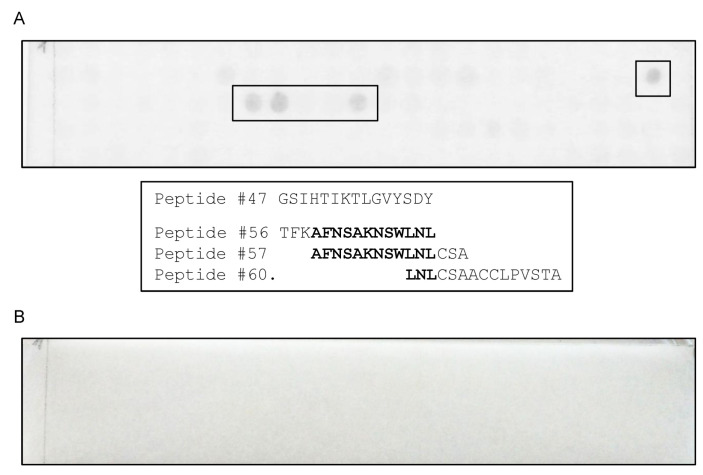
A cellulose membrane, containing 372 15-mere peptides covering the whole F13 protein, was used for epitope mapping. The scFv 3E2 (**A**) reacted with four peptide spots (No. 47, 56, 57 and 60). Two targets were mapped (139-GSIHTIKTLGVYSDY-153 and 166-TFKAFNSAKNSWLNLCSAACCLPVSTA-192). The minimal sequence essential for binding for the second epitope area was 169-AFNSAKNSWLNL-188. The secondary Ab, used as a negative control (**B**), did not bind to any spots.

A cellulose membrane, containing 372 15-mere peptides with 12 overlapping amino acids covering the whole F13 protein, was used for epitope mapping. The scFv 3E2 reacted to two different target regions, i.e., 139-GSIHTIKTLGVYSDY-153 and 166-TFKAFNSAKNSWLNLCSAACCLPVSTA-192 (Figure 4).

**Figure 5:**
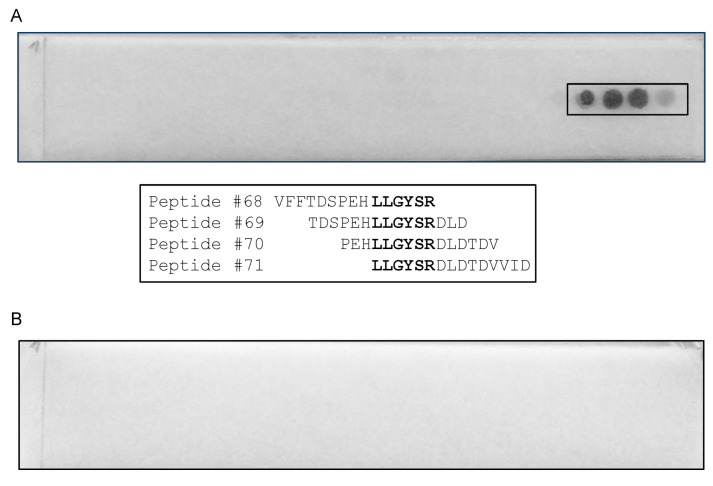
A cellulose membrane, containing 372 15-mere peptides covering the whole F13 protein was used for epitope mapping. MAb 15B6 (**A**) recognised four peptide spots (No. 68-71). The epitope was mapped to 202-VFFTDSPEHLLGYSRDLDTDVVID-225. The minimal sequence essential for binding is 211-LLGYSR-216. The secondary Ab, used as a negative control (**B**), did not bind to any spots.

The strongest signal was recorded for the area 169-AFNSAKNSWLNL-188. For the mAb 15B6, an epitope with the sequence 202-VFFTDSPEHLLGYSRDLDTDVVID-225 was identified (Figure 5), whereby the minimal sequence essential for binding was 211-LLGYSR-216.

**Figure 6:**
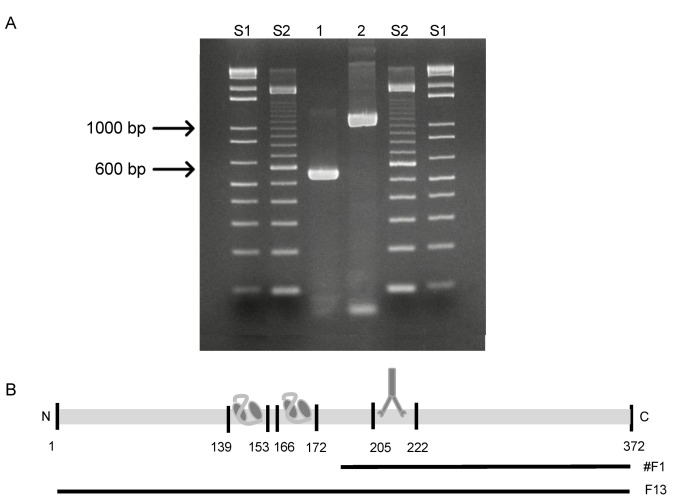
(**A**) Amplification of the 532 bp truncated fragment #1 of F13L_VACV_ (Lane 1) and the 1119 bp F13L gene (Lane 2). S1 and S2 are 1 kb and 100 bp ladders, respectively. (**B**) Schematic illustration of the F13 protein and the truncated fragment #F1. The epitope of the mAb 15B6 is located on the #F1, as well as on the whole F13 protein, while the antigenic sites of the scFv 3E2 are located only on the whole F13 protein.

To further confirm the epitope mapping results, a Western blotting analysis was performed. A truncated F13 fragment (#F1) starting at aa 197 with a total length of 532 bp was constructed (Figure 6A) to cover the identified epitope by the mAb 15B6 (Figure 6B).

**Figure 7:**
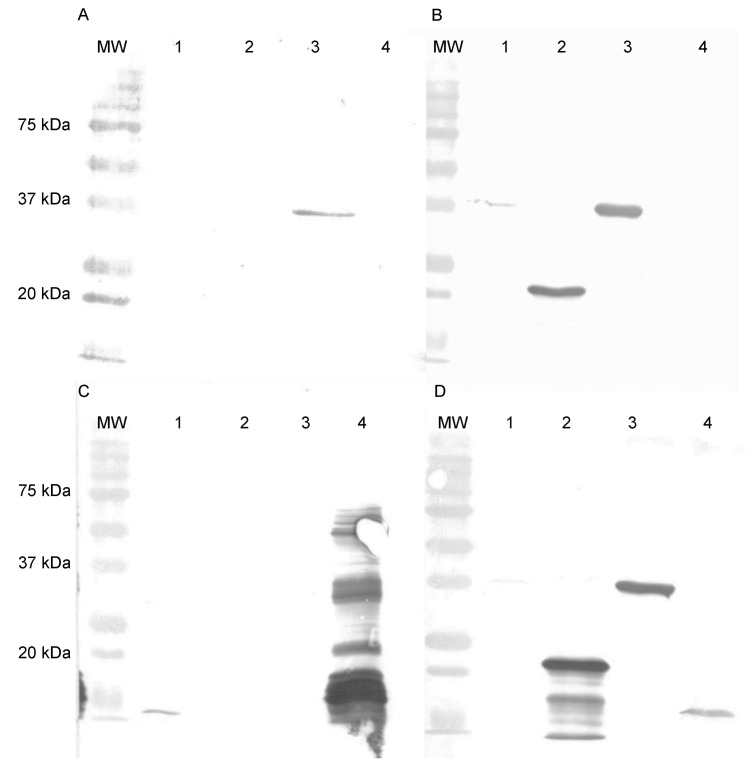
Western blotting analysis using VACV Elstree (Lane 1), #F1 (Lane 2), F13 (Lane 3) and A27 (Lane 4) proteins. The scFv 3E2 (**A**) detected the whole F13, while the mAb 15B6 (**B**) showed a reaction on the VACV Elstree, #F1 and the whole F13 protein. The control mAb 5B4/2F2 (**C**) was used and detected VACV Elstree and the recombinant A27 protein. The his-tag Ab (**D**) served as positive control and bound to all recombinant proteins. MW was Precision Plus Protein Standard (Bio-Rad).

The epitope mapping was verified by Western blotting analysis, whereby the mAbs 15B6 and 5B4/2F2, as well as an anti-his tag Ab, were used as controls (Figure 7). The scFv 3E2 only bound to the recombinant F13 protein (Lane 3), showing a band at 37 kDa (Figure 7A), whereas the mAb 15B6 showed a reaction with the recombinant fragment (Lane 2) at 22 kDa as well as with the whole F13 protein (Lane 3) and the VACV Elstree (Lane 1) at 37 kDa (Figure 7B). The mAb 5B4/2F2 recognised a 14 kDa on the recombinant A27 protein (Lane 4) and on the VACV Elstree (Lane 1) (Figure 7C). The anti-his-tag bound to all recombinant proteins and therefore confirmed the purity of the proteins. (Figure 7D).

### 3.4. Confocal Microscopy

**Figure 8:**
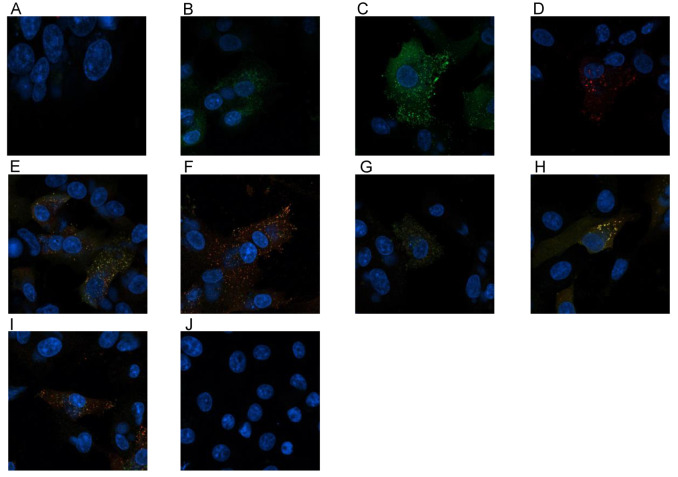
Confocal microscopy results of the scFv 3E2 (**A**), the mAb 15B6 (**B**), the mAb 5B4/2F2 (**C**), an anti-envelope rabbit immune serum (**D**), a mixture of the scFv 3E2 and the mAb 5B4/2F2 (**E**), a mixture of the mAbs 15B6 and 5B4/2F2 (**F**), a mixture of the scFv 3E2 and the mAb 15B6 (**G**), a mixture of scFv 3E2 and rabbit anti-envelope serum (**H**) and a mixture of mAb 15B6 and rabbit anti-envelope serum (**I**). All were able to penetrate the cell through permeabilisation to different extents. (**J**) is the negative control without VACV infection.

The VACV proteins F13 and A27 have both functions in virion wrapping. To investigate a believed interaction between them, a confocal study was done by infecting a confluent monolayer of Vero cells with 100 pfu of VACV Munich1 for 3 h. After fixing and permeabilisation of the cells, the Abs were incubated. Fluorescence was examined under a confocal laser scanning microscope. Penetrating the cell through permeabilisation was recognised for the following: scFv 3E2 (Figure 8A), the mAb 15B6 (Figure 8B), the mAb 5B4/2F2 (Figure 8C), an anti-envelope rabbit immune serum (Figure 8D), a mixture of the scFv 3E2 and the mAb 5B4/2F2 (Figure 8E), a mixture of the mAbs 15B6 and 5B4/2F2 (Figure 8F), a mixture of the scFv 3E2 and the mAb 15B6 (Figure 8G), a mixture of scFv 3E2 and rabbit anti-envelope serum (Figure 8H) and a mixture of mAb 15B6 and rabbit anti-envelope serum (Figure 8I). Various fluorescence signals were recorded. The scFv 3E2 (Figure 8A) showed the weakest signal, while the mAb 5B4/2F2 (Figure 8C) was the strongest. Interestingly, the mAb 15B6 and the rabbit anti-envelope immune serum bound to the same spot (Figure 8I).

### 3.5. Neutralisation Abilities In Vitro

**Figure 9:**
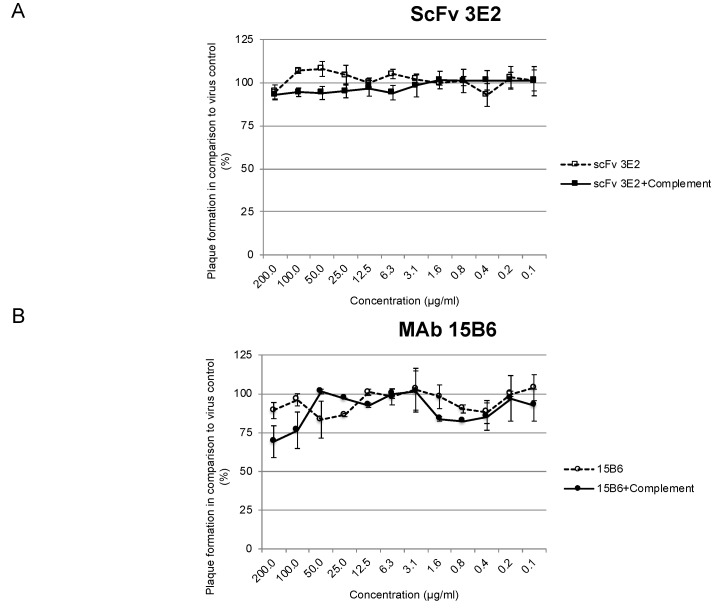
In vitro Plaque Reduction Neutralisation Test. The scFv 3E2 (**A**) and the mAb 15B6 (**B**) with and without complement did not neutralise the VACV Elstree.

The classical plaque reduction test (PRT) was performed in triplicates with a starting concentration of 200 µg/mL of the antibodies. The VACV-Elstree was neither neutralised by the scFv 3E2 (Figure 9A) nor by the mAb 15B6 (Figure 9) either in the presence or absence of 1% complement.

## 4. Discussion

In this study, we characterised a purified human anti-F13_VACV_ scFv, which was isolated from a human anti-OPXV-immunoglobulin library by phage display. The epitope of the scFv 3E2 was assigned to the 37 kDa F13 protein, which is the most abundant membrane protein of the EEV [28,29] and located within the TGN membrane [32]. F13 plays an important role in the wrapping progress and, therefore, in the EEV production. After EEV release, F13 is left on the inner side of the EEVs [56,57]. In Western blotting assays, the scFv 3E2 detected the recombinant F13 protein under denaturing and reducing conditions. However, the scFv 3E2 did not bind to its epitope on the whole VACV, which was expected due to the inner location of the protein [32,57]. These results were verified by ELISA, in which the scFv 3E2 bound to neither VACV Elstree nor VACV Munich1, too. Moreover, in the confocal microscopy study, the scFv 3E2 showed a weak signal only. The stronger binding of the mAb 15B6 to the virus can be explained because the mAb contains two antigen-binding sites, in contrast to the scFv 3E2, and due to a higher binding affinity.

For epitope mapping, a cellulose membrane, spanning 372 15-mere peptides with 12 overlapping amino acids covering the whole F13 protein was used. The scFv 3E2 reacted with two epitopes differently located on the F13 primary structure (139-GSIHTIKTLGVYSDY-153 and 169-AFNSAKNSWLNL-188). Another study shows that one antibody detected two almost identical epitopes of *Clostridium difficile* [58]. However, in the case of the scFv 3E2 target regions, no similarities between both regions were found. In Western blotting analysis, the scFv 3E2 binding resulted in a weaker band compared to the control mAb 15B6. Moreover, denaturation with SDS and heating reduced the binding of the scFv 3E2. Therefore, the scFv 3E2 is conformationally dependent. A similar result of a conformationally dependent herpes simplex mAb has been observed before [59]. Other discontinuous epitopes over a larger aa range were mapped on the D8 protein of VACV [60]. For the mAb 15B6, the epitope sequence of 211-LLGYSR-216 was identified. In predictions of the secondary structure of the F13 protein, ß-turns [61] were evident within the target region. Beta-turns generally lead to a high antigenicity [62]. Although several studies confirmed the neutralisation abilities of mAbs against OPXVs [39,63,64,65,66], the scFv 3E2 did not have any neutralising activity because of the inner localisation of the F13 protein [32]. However, scFvs can penetrate the cell, in contrast to full-size immunoglobulins, because of their small size [67,68]. In conclusion, the construction of recombinant scFv phage libraries is a promising strategy to generate engineered, target-specific human recombinant antibodies, which might help control any future outbreak of zoonotic OPXV infections.

## Data Availability

Raw data is available upon request.
